# Tau accumulations in the brains of woodpeckers

**DOI:** 10.1371/journal.pone.0191526

**Published:** 2018-02-02

**Authors:** George Farah, Donald Siwek, Peter Cummings

**Affiliations:** Department of Anatomy and Neurobiology, Boston University School of Medicine, Boston, Massachusetts, United States of America; Centre Hospitalier de l'Universite Laval, CANADA

## Abstract

Woodpeckers experience forces up to 1200–1400 g while pecking. It is assumed due to evolutionary adaptations, the woodpecker is immune to brain injury. This assumption has led to the use of the woodpecker as a model in the development of sports safety equipment such as football helmets. However, it is unknown at this time if the woodpecker brain develops neuro-trauma in relation to the high g-forces experienced during pecking. The brains of 10 ethanol preserved woodpeckers and 5 ethanol preserved red-winged black bird experimental controls were examined using Gallyas silver stain and anti-phospho-tau. The results demonstrated perivascular and white matter tract silver-positive deposits in eight out of the 10 woodpecker brains. The tau positive accumulations were seen in white matter tracts in 2 of the 3 woodpeckers examined. No staining was identified in control birds. The negative staining of controls birds contrasted with the diffuse positive staining woodpecker sections suggest the possibility that pecking may induce the accumulation of tau in the woodpecker brain. Further research is needed to better understand the relationship.

## Introduction

In the central nervous system of humans, the protein tau assists in the assembly and stabilization of neuronal microtubules. Accumulations of tau can be seen in conditions ranging from normal aging to various neurodegenerative diseases such as Alzheimer’s disease [1]. In the disease state, through a process yet to be understood, tau dissociates from the axons and become hyperphosphorylated to form insoluble neurofibrillary tangles [2]. It is not entirely clear if these aggregates are responsible for the symptoms associated with neurodegenerative diseases.

Tau accumulations have also been observed in association with chronic traumatic encephalopathy (CTE) [3]. CTE is theorized to be the end result of repetitive mild traumatic brain (mTBI) injury. Repeated concussions has been suggested as a cause of CTE in contact sport athletes.

Recently, the CTE center at Boston University reported CTE in brain tissue of 110 out of 111 former National Football League (NFL) players studied [4]. However, CTE is not unique to football and has been identified in the brains of athletes who play soccer, rugby and hockey [5].

The prevalence of CTE and relationship between mTBI and the subsequent development of CTE has yet to be fully established. Currently, the disease can only be diagnosed by post mortem analysis utilizing immunohistochemistry with antibodies directed against p-tau.

The most prevalent pathological changes thought to be diagnostic of CTE are focal accumulation of abnormal hyperphosphorylated tau (p-tau) within neurons and astroglia distributed around small blood vessels located at the depths of cortical sulci [6]. Other staining patterns considered to be supportive of CTE include pre tangles and neurofibrillary tangles in the superficial layers of the cortex and the hippocampus; neuronal and astrocytic aggregates in subcortical nuclei; and ‘thread-like’ and ‘dot-like’ axonal tau staining patterns [5, 7, 8]

Because of the theorized association between mTBI and CTE, the prevention of TBI in athletics has become an important area of research. Due to it's assumed resistance to neurotrauma, the woodpecker has become a model for the development of safety equipment such as football helmets and neck collars [9, 10].

The *Picidae* family of birds, which includes woodpeckers and sapsuckers, have several evolutionary anatomic adaptations to theorized to mitigate the enormous forces they experience while pecking. These include sharp beaks with upper and lower components which can move independently of each other while pecking and a long tongue that is capable of bracing the skull and brain during impacts. Other proposed protective adaptations include: thick neck muscles to dissipate force, and unique bony features of the skull [6]. It is assumed these evolutionary adaptations prevent neurotrauma in the woodpecker brain.

The majority of research using the woodpecker as a model for the development of safety equipment has focused on the biophysics of the woodpecker’s head. To date, only one paper has examined histologic sections of woodpecker brains in an effort to investigate the possible existence of brain injury in these birds. [11] This previous study utilized a modified Prussian blue stain (ferrocyanide) on the brains of two woodpeckers. Without describing their histologic findings, the authors concluded woodpeckers do not experience brain injury. Since its publication in 1976, this study has been sited in over 100 journal articles supporting the conclusion that woodpeckers do not incur brain injury in association with pecking behavior.

Despite the wide use of the 1976 paper, the neurobiological response of woodpeckers to repetitive head acceleration and deceleration is vastly unexplored. At this time, it is not known with any certainty if the brains of these animals experience neurotrauma in association with their pecking behavior. If so, the woodpecker may serve as an important animal model for the further study of CTE.

With the woodpecker model increasing in popularity as a potential source of protective equipment technology development, an in-depth and comprehensive study into woodpecker brain injury is warranted. [9] Given this, our group our group set out to determine if tau accumulations exist in the brains of woodpeckers.

## Materials and methods

Protocol doi: 10.17605/osf.io/cvupw

### Avian specimens and brain tissue collection

All the bird specimens used in this project were generously donated from museum collections (see acknowledgments). The woodpeckers (n = 10) studied were of various species (Table 1) To extract the brains, the feathers and skin covering the skull were removed. The skull cap was cut with a rotary tool from just above the orbits to just below the occiput in a circular fashion to remove the skull from the brain tissue below it. The plate of bone that protects the optic center of the brain, which is somewhat analogous to the human’s tentorium cerebelli, was removed using forceps. The brain was gently pried away from the the skull, taking great care around the frontal lobes and brainstem areas. Finally, an 11 blade scalpel was used to sever the brain stem right below the level of the pons. The extracted brains were then placed in 70% ethanol until tissue processing could occur.

**Table 1:** Summary of woodpecker specimens studied.

Species Name	Location Found	Gallyas Silver Stain	Immunohistochemistry
*Picoides pubescens*	St. Clair, MI	Negative	—
*Picoides pubescens*	Monroe, MI	Thread-like axonal streaks superficial and deep with tangles. Silver staining surrounding a few neuronal somas as well as dot-like deposits.	—
*Picoides pubescens*	Ann Arbor, MI	Thread-like axonal streaks superficially, with no tangles found. Perivascular deposits in superficial cortex	Anti-phospho-tau positive, thread-like streaks in an organized fashion, analogous location to the streaks seen in the silver stain of this specimen. Anti-GFAP positive star-like and tufted astrocytes.
*Sphyrapicus varius (*Juvenile)	Fairfield, ME	Thread-like axonal streaks superficially with dot-like deposits. No tangles or perivascular deposits.	—
*Picoides pubescens*	Harvard, MA	Negative	—
*Picoides pubescens*	Vienna, VA	Thread-like axonal streaks superficially with dot-like deposits. No tangles or perivascular deposits.	—
*Picoides pubescens*	Boston, MA	Localized axonal streaks at a superficial depth, limited deep streaks found. Focal perivascular as well as dot-like deposits.	—
*Phloeceatstes guatemalensis*	Mexico	Thread-like axonal streaks superficially and deep with no tangles. Numerous perivascular and dot-like deposits in superficial cortex.	Negative
*Colaptes auratus*	Lincoln, MA	Thread-like axonal streaks superficially and deep with no tangles or perivascular deposits.	—
*Dryocopus lineatus*	Canada	Thread-like axonal streaks superficially and deep with no tangles. Extensive perivascular and dot-like deposits in superficial cortex.	Anti-phospho-tau positive, thread like streaks deep in an organized, thread-like fashion, similar location to the streaks seen in the silver stain of this specimen. Anti-GFAP positive star-like and tufted astrocytes.

### Histological and immunohistochemical procedures

The woodpecker and red winged black bird brains were cut into gross tissue sections according to anatomic landmarks. Tissue processing was performed the same for all tissue samples according to standard paraffin-embedding processing procedures [12].

The Gallyas silver stain was performed in accordance to a previous published methodology with some minor alterations at room temperature with slight agitation [13]. Experimental and control slides were sectioned to 15μm onto slides, deparaffinized, and rehydrated back to water. The slides were then placed in a 0.25% potassium permanganate for 15 minutes. Then, slides were incubated in 2% oxalic acid for 5 minutes. Following the oxalic acid incubation, the slides were rinsed in dH_2_O for 5 minutes before being placed in a 0.4% lanthanum nitrate/2% sodium acetate blocking solution for one hour. After the blocking step, the slides were rinsed again in dH_2_O for one minute before going into a 4% sodium hydroxide/10% potassium iodide/0.035% silver nitrate (added in that order) solution for four minutes. The slides were immediately placed into a 0.5% acetic acid for three, one minute rinses before being placed into developer. The slides were then put into 1% acetic acid for three minutes and then placed into dH_2_O. Mayer’s hematoxylin counterstain (Scytek, #HAQ999) was used by placing the silver stained sections into the stain for two minutes before a quick rinse in dH_2_O. The slides were differentiated with 0.1% sodium bicarbonate bluing agent until desired color was achieved. Slides were then dehydrated using the standard ethanol gradient and Histoclear before being coverslipped.

The immunohistochemical staining was achieved using a hybrid of previously published techniques. [14] The tissue was cut at 25μm and each section was placed into a 2cm in diameter steel-wire mesh container. The tissue slices were deparaffinized and rehydrated to water before entering the antigen retrieval step. Antigen retrieval was done by submerging the mesh containers into filtered 1x Tris/EDTA buffer pH 9 with 0.05% Tween 20 at 90°C for 20 minutes. After the 20-minute incubation, the mesh containers were then placed into filtered 1x TBS buffer pH 7.4 with 0.025% Triton X-100 and rinsed twice for five minutes each. After the second rinse, the mesh containers were removed and incubated in filtered 10% goat serum/1x TBS buffer pH 7.4 block for two hours. After incubation, tissue sections were removed from the mesh containers and placed into individual sterilized petri dishes containing antibody in sterile and filtered 1% goat serum 1x TBS buffer pH 7.4 overnight at 4°C with gentle agitation. The antibodies used were anti-phospho-tau S262 rabbit polyclonal (5μg/mL; Abcam, ab64193; Cambridge, MA) and anti-glial fibrillary associated protein rabbit polyclonal (5μg/mL; Bioss, bs-0199R; Woburn, MA). Following overnight incubation, the sections were removed from the primary antibody and placed back into the mesh containers to be rinsed with 1x TBS with 0.025% Triton X-100 twice, each for five minutes. The mesh containers were then placed into 0.3% sodium hydroxide 1x TBS buffer pH 7.4 for 15 minutes. After the sodium hydroxide incubation, the mesh containers were once again rinsed for three minutes in 1x TBS buffer pH 7.4 with 0.025% Triton X-100. The sections within the mesh containers were removed once again, and placed into small, sterile petri dishes containing horseradish peroxidase secondary antibody (1μg/mL; Abcam, ab6721; Cambridge, MA) in 1x TBS buffer pH 7.4 for one hour at room temperature. After the hour incubation, the sections were placed back into their mesh containers and rinsed twice, for five minutes each, in 1x TBS buffer pH 7.4. The sections were then ready for the 3,3'-diaminobenzidine (DAB) reaction. The DAB chromagen (Biocare Medical; DB801R; Concord, CA) reaction was carried out under a microscope until adequate staining was achieved. Sections were then immediately placed back into their mesh containers and rinsed with dH_2_O for five minutes. Following the dH_2_O rinse, the sections were counterstained with Mayer’s hematoxylin counterstain (Scytek, #HAQ999) for two minutes before a quick rinse in dH_2_O. The sections were then differentiated with 0.1% sodium bicarbonate bluing agent until desired color was achieved. After another quick rinse in dH_2_O, the sections were dehydrated using the standard ethanol gradient and Histoclear while in the mesh containers. After the last Histoclear incubation, the sections were removed from their mesh containers and free-floated into a large crystallization dish containing Histoclear. The sections were coaxed onto clean glass slides with fine tipped forceps. The slides were then cover slipped with mounting medium and placed onto a 37°C warming plate overnight.

## Results

### Gallyas silver stain

Because Gallyas stain has a high degree of sensitivity and specificity for neurofibrillary tangles and axonal injury, it was utilized to detect the presence of neuronal and/or white matter tract damage throughout the entire woodpecker brain.

A section of human cortex with confirmed Alzheimer’s disease was used as a positive staining control while red-winged black birds’ brains (n = 5) were used as experimental controls for all staining methods.

**Fig 1:**
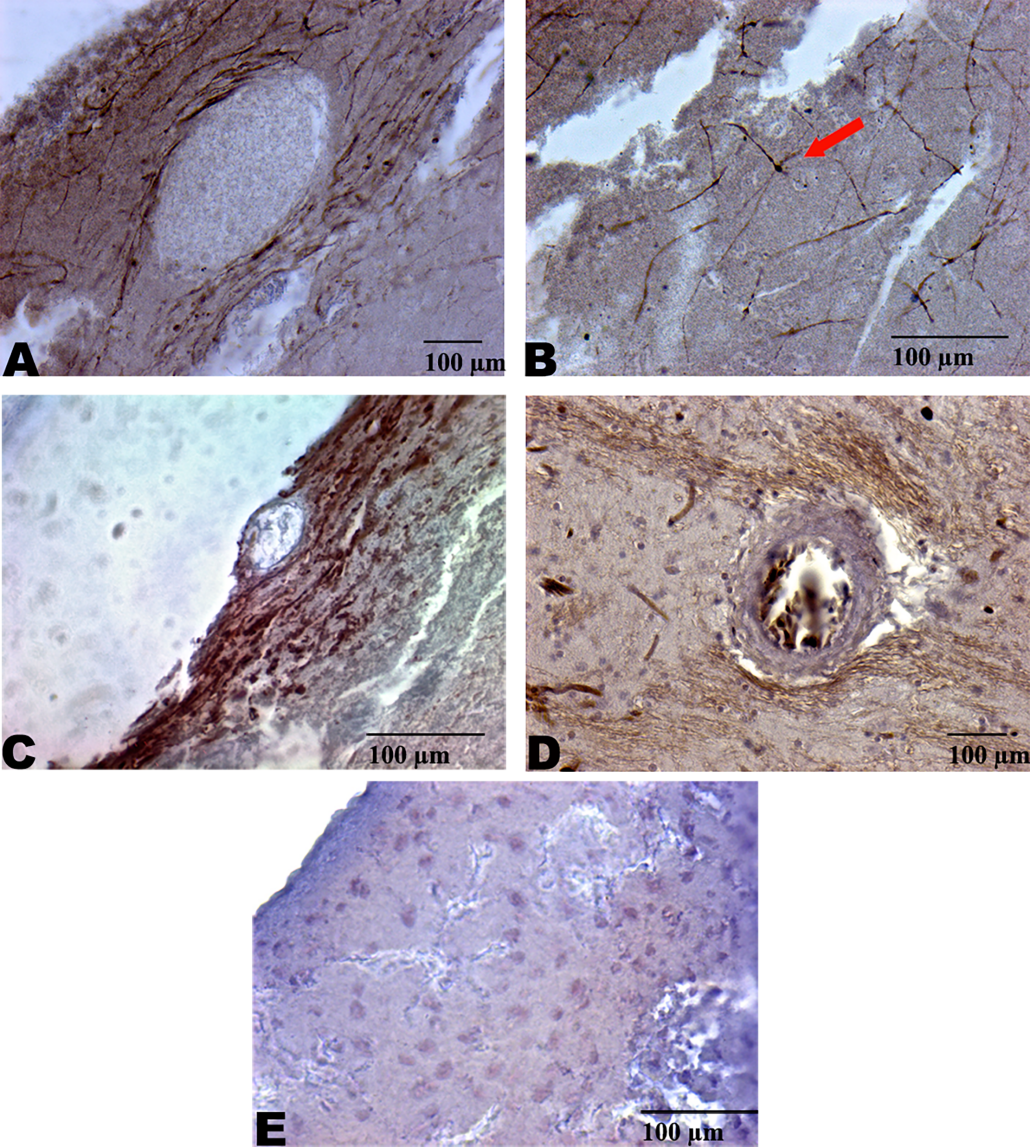
Perivascular and axonal track pathology of *Dryocopus lineatus* and *picoides pubescens*. Perivascular Gallyas silver positive pathology in the cortex of the frontal lobe (A). Damaged axonal tracts (B) with axonal swellings (arrow) in the subcortical white matter of the frontal lobe. Subpial perivascular staining (C) of the frontal lobe. Perivascular silver positive staining in (D) in a superficial region of the frontal cortex. Experimental controls (E).

Positive silver accumulations were identified in 8 out of the 10 woodpeckers studied. Several patterns of positive Gallyas staining were identified in the woodpecker population including: focal perivascular deposits, which were mostly subpial, (Fig 1A, 1C and 1D); focal whole astrocyte staining (Fig 1B); dot-like staining within axonal tracks; and wide-spread thread-like axonal track staining in deep white matter tracks. The majority of the observed silver-positive staining patterns were identified in the frontal pole of the brain. Very rare staining was detected in the occipital region, and no staining was seen in the cerebellum.

**Fig 2:**
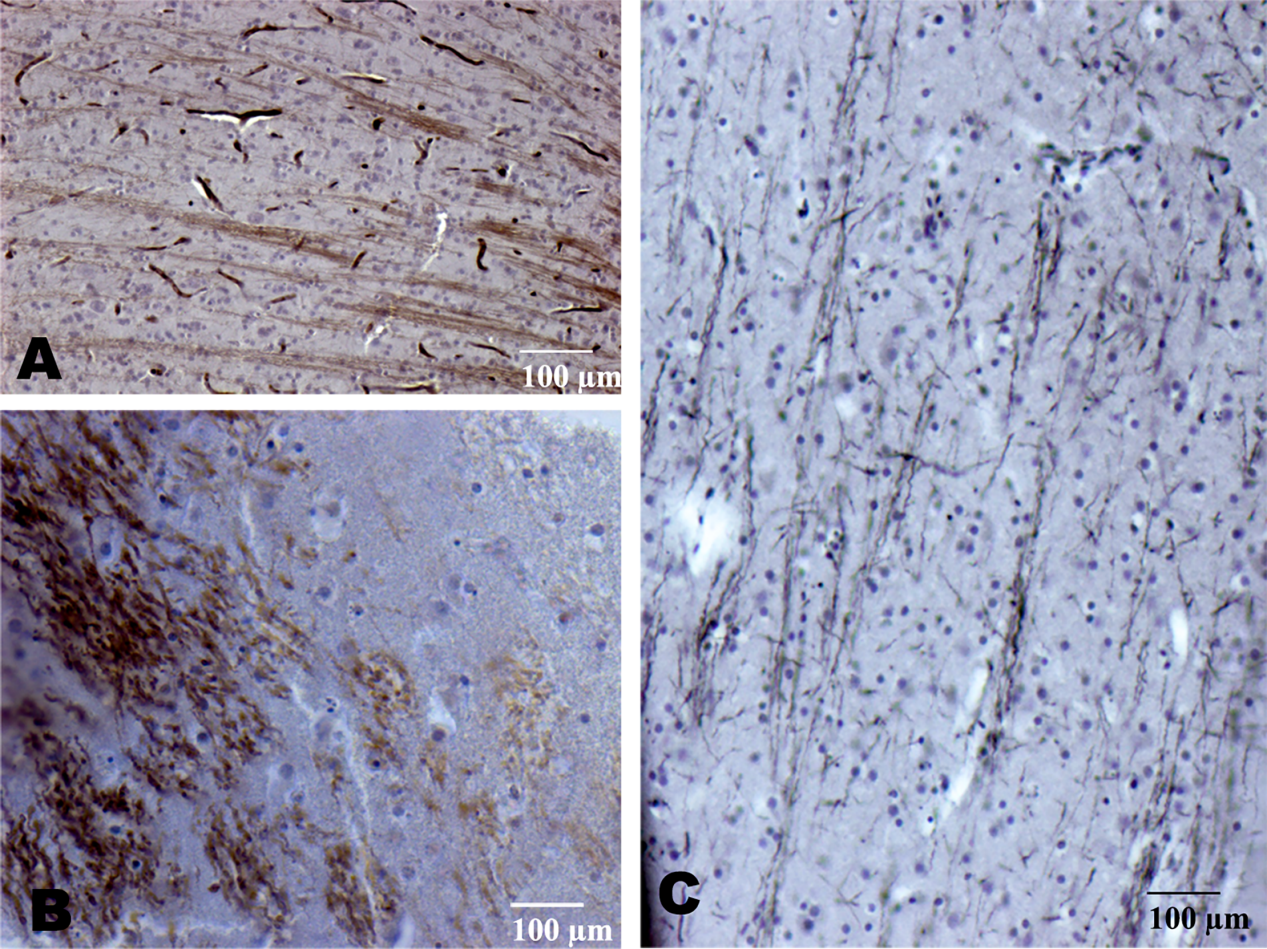
Axonal tract pathology of *Dryocopus lineatus* and *Picoides pubescens*. Gallyas silver positive axonal tract staining in the corpus callosum (A) and the mediolateral central gray area of the midbrain (B and C) in the woodpecker brain (A) [15].

Focal perivascular silver-positive deposits were found in 40% of the woodpeckers. The most abundant positive silver staining pattern was “thread-like” axonal white matter tracts of the frontal lobe, which appeared in 80% (n = 10) of the woodpeckers studied (Fig 2).

No positive Gallyas staining was observed in the control birds (n = 10).

### Immunohistochemistry

Following the analysis of the Gallyas silver stain, we proceed to immunohistochemical verification of the suspected tau accumulations throughout the entire woodpecker brain. Samples were stained for phosphorylated tau (S262).

Due to the poor preservation of the woodpecker tissue, successful immunohistochemistry was accomplished in only 3 birds; the remainder of the tissue samples degraded during processing. Attempts to alter the immunohistochemistry methodology proved to not help with the degradation of the tissue samples. Immunohistochemistry was performed on all control birds (n = 5).

**Fig 3:**
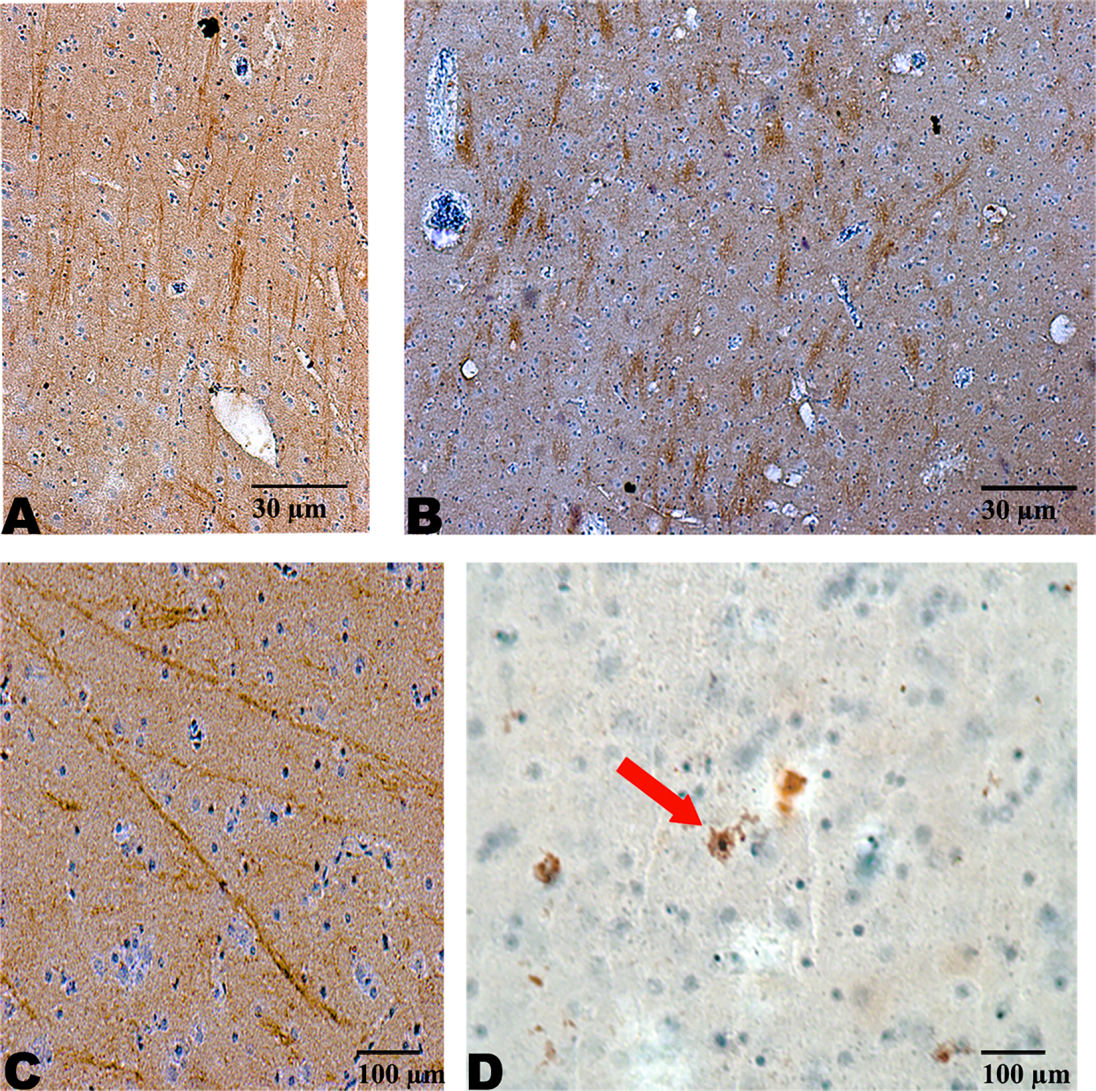
Anti-phospho-tau immunostaining in *Dryocopus lineatus*. Tau-positivity in the midbrain (A and B) and the corpus callosum (C) of the *Dryocopus lineatus* brain. The axonal tract staining demonstrates a thread-like pattern, similar to that seen with Gallyas sliver staining (Fig 2). Occasional intracellular tau-accumulations were identified within neurons (D).

In the woodpecker specimens where tau immunostaining was possible, tau-positive accumulations were identified in the same regions highlighted originally by the Gallyas silver stain. The morphology of astrocytes identified by GFAP were used to determine some of the cells staining with tau were in fact astrocytes. This assisted in confirming the silver-positive accumulations identified were in fact comprised of tau protein. Specifically, tau immunostaining demonstrated perivascular deposits, whole astrocyte staining, and ‘thread-like’ axonal track staining in 2 of the 3 woodpecker brains evaluated (Fig 3).

No positive immunostaining was identified in any of the control birds.

**Fig 4:**
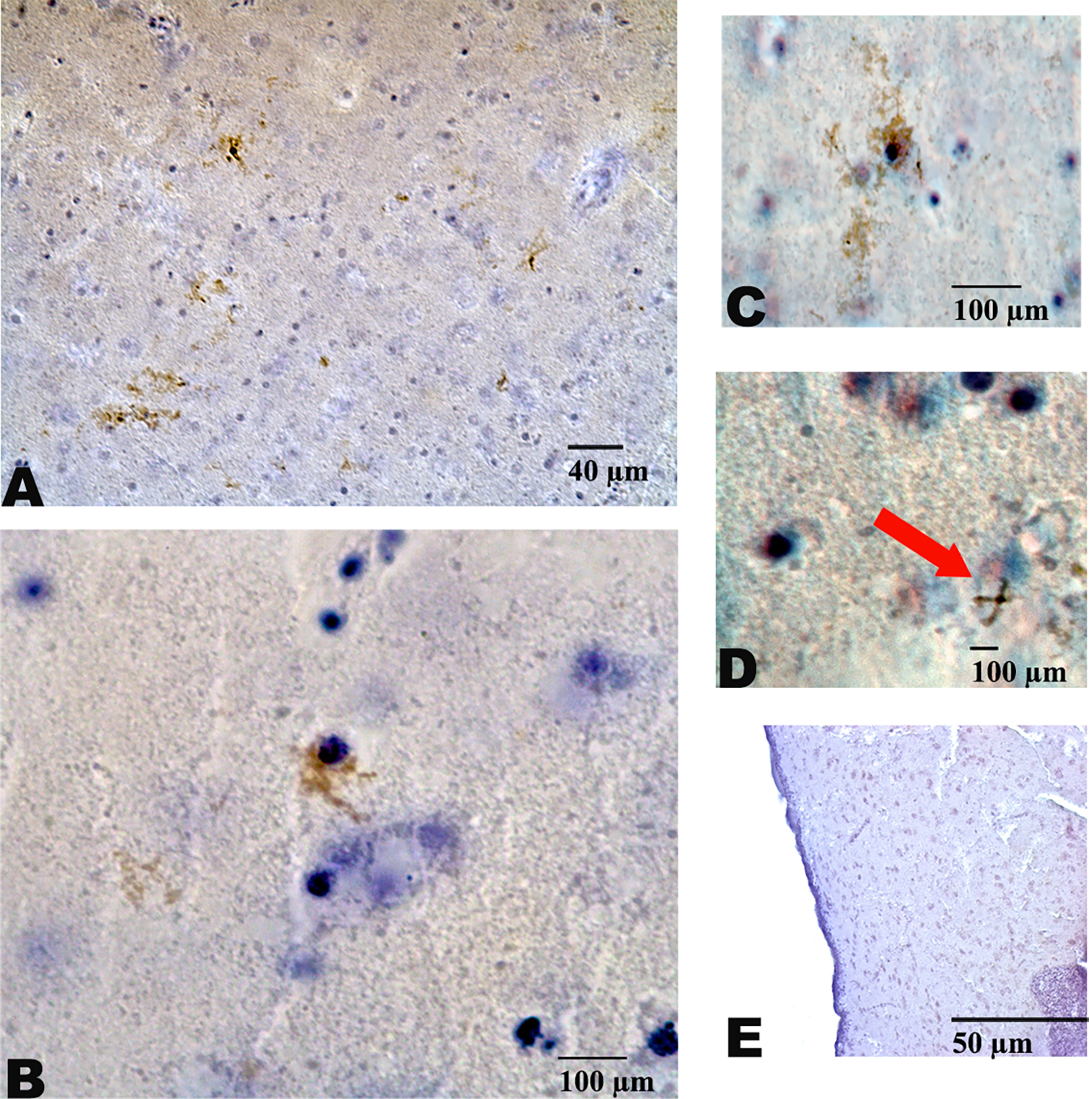
Anti-GFAP immunostaining in *Dryocopus lineatus*. Immunostaining with GFAP demonstrated rare GFAP-positive astrocytes. Astrocyte morphology included thorn-shaped (A), typical star-like (B) and tufted (C). Tau immunohistochemistry demonstrated rare tau accumulations in cells morphologically consistent with astrocytes within the grey matter (D, arrow).

Tau immunohistochemistry was successfully completed in three woodpecker brains. Two of the three woodpecker brains demonstrated wide-spread thread-like axonal track staining with tau. We observed positive GFAP-staining in these same two birds (Fig 4). One woodpecker failed to demonstrate positive sliver and tau staining. This same bird also showed negative GFAP immunohistostaining. Interpreted collectively, this suggests that the tau accumulations we identified are pathological.

In summary, focal subpial perivascular deposits; focal whole astrocyte staining; dot-like staining within axonal tracks; and wide-spread thread-like axonal track staining in white matter tracks were identified in the frontal poles of 80 percent of the study population. Strikingly, no staining was identified in the control bird population.

These observations were confirmed by a board-certified neuropathologist.

## Discussion

To date, there have been no histologic studies exploring the potential existence of neurotrauma in woodpeckers. While it is unknown if the forces associated with pecking behavior could result in traumatic brain injury, it is interesting that the majority of the woodpecker specimens in our study displayed focal silver-positive deposits, some of which were confirmed to be tau by immunohistochemistry, while no staining was observed in the control birds.

The anatomic locations and staining patterns of the lesions identified in the brains of woodpeckers shares some similarities to human CTE. In humans, CTE is most prominent in the frontal and temporal lobes of the brain with a spectrum of tau deposition patterns including focal perivascular staining, astrocytic inclusions, ‘thread-like’ and ‘dot-like’ axonal staining patterns (5, 7). In the woodpecker, we identified similar focal perivascular staining, astrocytic inclusions, ‘thread-like’ and ‘dot-like’ axonal staining patterns which were confined to the frontal lobe of the brain. The woodpecker brain lacks the gyri and sulci seen in the human brain. Because of this, we could not evaluate for lesions located at the depth of sulci, as seen in human CTE.

The prominent frontal and temporal anatomic locations of CTE lesions are thought to be due to the distribution of force experienced in head collision [14]. In the woodpecker, much of the force of pecking is thought to be dissipated through the frontal regions of the skull and brain, as well. Therefore, it is not surprising to see potential areas of injury limited to this anatomic location in woodpecker brain.

In the human brain, tau accumulations are also known to occur as part of the normal aging process. Though it cannot be entirely ruled out, age-related tau accumulation in the woodpecker is an unlikely explanation for our findings. Our study population included one juvenile woodpecker (*Sphyrapicus varius)* which demonstrated the full spectrum of tau accumulations observed in the majority of the adult population suggesting the tau accumulations seen in our study might not be age-related. This notion is further supported by the lack of observable staining in the control population which was comprised of entirely adult birds.

Given the complete lack of staining in the control population and the unlikely scenario of age-related changes, our findings suggest there might be an association between repetitive pecking behavior and tau accumulations in the woodpecker population.

There are several limitations to this study. It cannot be concluded at this time the if the histologic changes identified in our study are the direct result of the repeated, high force pecking woodpeckers endure everyday. The limited number of woodpeckers (n = 10) and control birds (n = 5) utilized in this study are insufficient to established a correlation between pecking behavior and subsequent neurotrauma.

It is not known from our study whether the tau accumulations are pathological or result in behavioral changes in woodpeckers. However, our findings of silver and tau accumulations solely in pecking birds warrant further investigation into this possibility.

There are numerous anatomic differences between the skulls and brains of woodpeckers. It may be that the anatomic adaptations of the woodpecker produce stress in regions of the brain in different locations than humans. Further research is necessary to understand how our findings can be translated to the human population.

Due to the increased tau deposition in our woodpecker population, the brains of woodpeckers are an important area for future research. Further studies are needed to determine what iso-forms of tau are being deposited in the woodpecker brain and if these deposits are pathological. The continued study of the response of the woodpeckers’ brain to pecking is necessary to assure current head protection technology based on the woodpecker model is providing adequate protection in athletes. Our findings also suggest the woodpecker may be a suitable animal model for the further study of CTE.
